# Wound Healing: Biologics, Skin Substitutes, Biomembranes and Scaffolds

**DOI:** 10.3390/healthcare2030356

**Published:** 2014-09-10

**Authors:** Krishna S. Vyas, Henry C. Vasconez

**Affiliations:** Division of Plastic Surgery, Department of Surgery, University of Kentucky, Kentucky Clinic K454, 740 South Limestone, Lexington, KY 40536, USA; E-Mail: krishnavyas@uky.edu

**Keywords:** biologics, skin substitutes, biomembranes, scaffolds, wound healing

## Abstract

This review will explore the latest advancements spanning several facets of wound healing, including biologics, skin substitutes, biomembranes and scaffolds.

## 1. Introduction

The healing of wounds is a complex process that involves the activation and synchronization of intracellular, intercellular and extracellular elements, including coagulatory and inflammatory events, fibrous tissue accretion, deposition of collagen, epithelialization, wound contraction, tissue granulation and remodeling [1]. This process occurs via activation of local and systemic cells to restore tissue integrity through regeneration and scar formation, and often these cumulative processes result in satisfactory repair of damaged sites. Disruptions caused by tissue loss, inadequate blood flow, and comorbid disease states can lead to chronic wounds that are difficult to manage [2]. There are many strategies that have been applied to the treatment of wounds in the past. Early on, these were based on empirical deduction and unsubstantiated determinations. Although there was a general resistance to new concepts and modalities that impeded progress, advancements in the treatment of wounds have, nevertheless, evolved [3]. Over the past two decades, advancements in the clinical understanding of wounds and their pathophysiology have commanded significant biomedical innovations in the treatment of acute, chronic, and other types of wounds. This review will explore the latest advancements spanning several facets of wound healing, including biologics, skin substitutes, biomembranes and scaffolds.

## 2. Biologics for Wound Healing

### 2.1. Description

Biologic wound healing therapies are those that are intended to facilitate the re-establishment of the innate repair mechanisms, and may involve the application of active biological agents, such as plant-derived active biomolecules which exhibit antioxidant, antimicrobial, or anti-inflammatory attributes. Biologic dressings prevent evaporative water loss, heat loss, protein and electrolyte loss, and contamination. They also permit autolytic debridement and develop a granular wound bed. Biological skin equivalents, epidermal growth factors, stem cell therapies, and tissue engineering might also be utilized [2]. 

### 2.2. Mechanisms and Indications

Monoterpenes represent an extensive and varied family of naturally occurring terpene-based chemical compounds that comprise the majority of essential oils. These compounds exhibit anti-inflammatory, antibacterial, and antioxidant attributes [4,5]. The primary mechanisms proposed for various monoterpenes encompass: antimicrobial activity (inhibition of microorganism ribonucleic acid (RNA) and protein biosynthesis); anti-inflammation (lowers the generation of interleukin 6 (IL-6) and tumor necrosis factor alpha (TNF-α) in mast cells, inhibition and alteration of leukotriene C4 (LTC4) release and thromboxane B2 (TXB2) release, respectively); antioxidation (inhibits the production of ultraviolet B (UVB)-induced free radicals photoprotective effects and oxidative stress); fibroblast growth and macrophage migration inhibitory factor (MIF) effects. The anti-inflammatory action of the monoterpenes is often correlated to their wound-healing effects. Monoterpenes include compounds such as borneol, thymol, α-terpineol, genipin, aucubin, *d*-Limonene and sericin that have either direct or indirect activities in wound healing. Although monoterpenes are poorly studied in the context of wound healing, studies suggest that they are promising for the treatment of chronic wounds (Table 1).

Mai *et al.* [6] investigated the ointment Sulbogin^®^ (marketed as Suile^TM^), comprised of borneol (a bicyclic monoterpenoid alcohol), bismuth subgallate and Vaseline^®^, and found it to hasten excision wound closure in adult male *Sprangue-Dawley* rats. Although the specific mechanism remains elusive, it is thought that bismuth subgallate may induce macrophages to secrete growth factors to facilitate wound healing. It was found to decrease the lesion area, enhance granulation tissue formation and re-epithelialization, initiate the proliferation of collagen via the activation of fibroblasts, accelerate the reestablishment of blood vessels, and restrict the formation of nitric oxide (NO) [4,6]. 

The monoterpenoid phenol, thymol, demonstrates multiple beneficial bioactivities toward the healing of wounds. These attributes encompass the modulation of prostaglandin synthesis [7], imparting anti-inflammatory effects in neutrophils, the inhibition of myeloperoxidase activity and a decreased influx of leukocytes [8,9], positive antioxidant effects on docosahexaenoic acid (an omega-3 fatty acid) concentrations [10], the prevention of lipid autoxidation [11] and formation of toxic elements via the stimulation of reactive nitrogen species [12], and antimicrobial activity [13,14]. The capacity of thymol for direct wound healing involves its being correlated with elevated concentrations, in the central nervous system, of macrophage MIF, as well as enhanced anti-inflammatory related tissue granulation. Furthermore, it influences collagen synthesis and fibroblast metabolism, leading to augmented fibroblast growth *in vitro* [9].

**Table 1:** Monoterpenes in wound healing.

Monoterpene	Company (FDA Approval)	Composition	Mechanism	Clinical Trials
Sulbogin^®^ (Suile^TM^) ointment wound dressing	Hedonist Biochemical Technologies Co, Taipei, Taiwan (2001, 2003)	0.7% borneol, 4.5% bismuth subgallate, Vaseline^®^	bismuth subgallate induces macrophages to secrete growth factors to facilitate wound healing [6] decreases lesion area, enhances granulation tissue formation and re-epithelialization, initiates proliferation of collagen via the activation of fibroblasts, accelerates reestablishment of blood vessels, restricts the formation of nitric oxide [4]	Indicated for first- and second-degree burns, partial-thickness wounds, donor sites and abrasions. In a study evaluating the effect of bismuth subgallate on biopsy punch wounds on Wistar rats, bismuth subgallate had a statistically significant improvement in the area of ulceration (day 1), distance between epithelial edges (day 4), and area of granulation tissue (day 7, 11, 18) compared to control. No significant histological differences were identified between the test and control [15]. A study of adult male rats with full-thickness wounds were evaluated using the treatment bismuth and borneol, the major components of Sulbogin^®^ with control treatment flamazine. The experimental treatment decreased the wound lesion area, increased granulation tissue formation and re-epithelialization [6].
thymol	N/A	monoterpenic phenol which is usually found in thyme oil	modulates prostaglandin synthesis [7]; anti-inflammatory; inhibits myeloperoxidase activity [8,9]; oxidant effects on docosahexaenoic acid [10]; prevents lipid autoxidation [11] and formation of toxic elements via the stimulation of reactive nitrogen species [12]; enhances collagen synthesis and fibroblast metabolism [9]; antimicrobial; anesthetic [16]	Wounds dressed with collagen-based containing thymol films showed significantly larger wound retraction rates at 7 and 14 days, improved granulation reaction, and better collagen density and arrangement [9]. Gelatin films impregnated with thymol have antioxidant and antimicrobial properties against *Staphylococcus aureus*, *Bacillus subtilis*, *Escherichia coli*, and *Pseudomonas aeruginosa* [17].
α-terpineol	N/A	monoterpene alcohol derived from pine and other oils	inhibits generation of prostaglandin-endoperoxide synthase [18], COX-2 [19], IL-1β [20], IL-6 [21], NF-κB [20], TNF-α and NO production [21]; increased expression of IL-10; inhibits neutrophil influx [22]; antimicrobial [23]; antifungal [24]	No clinical trials in wound healing.
genipin	N/A	fruit extract aglycone derived from iridoid glycoside	crosslinking agent [25,26]; antioxidant [27]; anti-inflammatory [28]; stimulates NO production; inhibits lipid peroxidation; elevates potential of mitochondrial membranes; elevates secretion of insulin; increases ATP levels; closes K_ATP _channels [29]	No clinical trials in wound healing. Genipin hydrogels [30], nanogels [31], and genipin cross-linked scaffolds [32] have potential application in skin tissue engineering [33] and wound dressings [34,35,36] and demonstrate excellent biocompatibility and low cytotoxicity in scaffolding models [37,38]. In biomaterials studies, genipin-crosslinked gels enhance fibroblast attachment [39] and vascularization of engineered tissues [38,40] and exhibit bacterial inhibition [41] Genipin-crosslinked gelatin-silk fibroin hydrogels have been shown to induce pluripotent cells to differentiate into epidermal lineages [42]. Genipin as a crosslinking agent is also utilized in controlling drug delivery in multiple systems [43].
aucubin	N/A	iridoid glycoside found in plants	anti-inflammatory [44], antimicrobial, antioxidant, chemopreventive agent	No clinical trials in wound healing. In a study of male mice with full-thickness buccal mucosal oral wounds, 0.1% aucubin-treated mice demonstrated earlier re-epithelization and matrix formation and decreased numbers of inflammatory cells compared to saline-treated controls at 1, 3, and 5 days, suggesting utility of topical aucubin in oral wound healing [45].
*d*-Limonene	N/A	orange-peel derived terpene d-Limonene	anti-angiogenic, anti-inflammatory; decreases systemic cytokines; inhibits expression of endothelial P-selectin	No clinical trials in wound healing. Topical d-Limonene and its metabolite perillyl alcohol were tested in murine models of chemically-induced dermatitis and mechanical skin lesions. Both significantly reduced the severity and extent of chemically-induced dermatitis. Lower levels of the inflammatory cytokines IL-6 and TNF-α, reduced neovascularization, and lower levels of P-selectin expression were observed in both models. Both d-Limonene and perillyl alcohol demonstrated anti-inflammatory effects in wound healing. Together, these effects contribute to the wound healing effects of d-Limonene [46]. Nanophyto-modified wound dressings with limonene are resistant to Staphylococcal and Pseudomonal colonization and biofilm formation compared to uncoated controls [47]. Topical limonene and other terpenes can increase permeation of silver sulphadiazine by increasing its partitioning into eschars. Burn wound antimicrobial therapy may be improved through the use of terpenes [48].
sericin	N/A	protein created by silkworms (*Bombyx mori*)	stimulates migration of fibroblasts; generates collagen in wounds, leading to activation of epithelialization; anti-inflammatory; initiates propagation and attachment of skin fibroblasts and keratinocytes	Double blinded randomized controlled trial (RCT) of 65 burn wounds of greater than 15% total body surface area (TBSA) were randomly assigned to either control (silver zinc sulfadiazine cream) or treatment (silver zinc sulfadiazine cream with sericin cream at a concentration of 100 μg/mL). Time to complete healing was significantly shorter for the treatment group (22.42 ± 6.33 days) compared to the control group (29.28 ± 9.27 days). No infections or adverse reactions were found in any of the wounds [49]. A clinical study on silk sericin-releasing wound dressing was compared to the wound dressing Bactigras^®^ in a clinical trial in patients with split-thickness skin graft (STSG) donor sites. The sericin dressing was less adhesive to the wound and potentially less traumatic. Wounds treated with the silk sericin dressing exhibited significantly faster rates to complete healing (12 ± 5.0 days compared to 14 ± 5.2 days) and significantly reduced pain during the first four days post-operatively [50]. In rat models, silk sericin dressing also demonstrated accelerated wound healing and greater epithelialization and type III collagen formation in full-thickness wounds [51,52,53] Several animal studies conclude that sericin promotes the wound healing process without causing inflammation [54]. Sericin treated full-thickness skin wounds in rats demonstrated less inflammation, greater wound size reduction and shorter mean time to healing compared to control (betadine treated full-thickness skin wounds). Examination after 15 days of 8% sericine treatment revealed complete healing, increased collagen formation, and no ulceration compared to cream base-treated wounds which demonstrated inflammatory exudates and ulceration [55]. 3D hydrogels [56] and cultured fibroblasts and keratinocytes on three-dimensional sericin matrices can potentially be used as skin equivalents in wound repair [57]. Sericin/chitosan composite nanofibers demonstrate wide spectrum bactericidal activity [58]. Sericin enriched wound dressings represent significant promise in wound healing biologics [35,59,60].

The monoterpenoid alcohol, α-terpineol conveys its wound healing [61] and anti-inflammatory activities via the inhibition of the generation of prostaglandin-endoperoxide synthase enzymes [18], cyclooxygenase-2 (COX-2) [19], interleukin-1 beta (IL-1β) [20] and IL-6 cytokines [21], nuclear factor kappa-light-chain-enhancer of activated B cells (NF-κB) [20], TNF-α and NO production [21]. Increased expression of the anti-inflammatory cytokine interleukin 10 (IL-10) is also observed. Additionally, it exhibits inhibitory effects on neutrophil influx [22], as well as robust antimicrobial [23] and antifungal activities [24]. Significant activity in tissue/scar formation is also observed with α-terpineol [61]. 

Cross-linkers are one of the many factors that affect the mechanical and biological properties of scaffolds used in tissue engineering. The iridoid (a secondary monoterpenoid metabolite) compound genipin may serve as a biocompatible crosslinking agent that imparts minimal cytotoxicity [25,26]. Additionally, it is an antioxidant [27] and anti-inflammatory that stimulates the generation of NO while inhibiting lipid peroxidation [28]. It also serves to elevate the potential of mitochondrial membranes, to elevate the secretion of insulin, to increase adenosine triphosphate (ATP) levels and to close potassium channels (K_ATP_) [29], among other positive effects in wound healing [36,62]. Aucubin (an iridoid glycoside) was found to have beneficial pharmacological activities on a number of fronts, encompassing dermal wound healing [44,45,63], and capacities as an anti-inflammatory [44], antimicrobial [64], and antioxidant [65]. In addition to various specific biochemical effects, it also shows promise as a non-cytotoxic chemopreventive agent [66]. 

D’Alessio *et al.* [46] revealed that the prototype monoterpene *d*-Limonene in combination with its metabolite perillyl alcohol, which is derived from orange-peel, exhibited considerable anti-angiogenic, anti-inflammatory properties, epidermal repair and wound healing effects in murine models. These compounds also lowered the generation of systemic cytokines and inhibited the expression of endothelial P-selectin. Topical treatment resulted in more rapid and improved wound closure.

Aramwit *et al.* [49] revealed that a protein derived from the silkworm cocoon called silk **sericin** acted to enhance the capacity for wound (second-degree burns) healing when incorporated into a common silver zinc sulfadiazine antimicrobial cream. At a concentration of 100 μg/mL, sericin was shown to stimulate the migration of fibroblasts. Siritientong *et al.* [35] discovered that silk sericin had the capacity to generate collagen in wounds, which led to the activation of epithelialization. Further, it served to reduce inflammation [67] and to initiate the propagation and attachment of human skin fibroblasts and keratinocytes [55,68,69].

### 2.3. Contraindications

Contraindications for biologics such as the monoterpenes are low. Acute toxicity of the monoterpenes is low via the oral and dermal routes of exposure in animal models [70].

## 3. Skin Substitutes for Wound Healing

### 3.1. Description

Skin substitutes are tissue-engineered products designed to replace, either temporarily or permanently, the form and function of the skin. Skin substitutes are often used in chronic, non-healing ulcers, such as pressure ulcers, diabetic neuropathic ulcers and vascular insufficiency ulcers. These wounds contribute to substantial morbidity such as increased risk for infection, limb amputation, and death. Skin substitutes have the potential to improve rates of healing and reduce complications in a variety of other skin wounds including, but not limited to, wounds from burn injuries, ischemia, pressure, trauma, surgery and skin disorders. Skin substitutes are also used in patients whose ability to heal is compromised and in situations where skin coverage is inadequate. Goals for treating acute and chronic wounds with skin substitutes are to provide temporary coverage or permanent wound closure, to reduce healing time, to reduce post-operative contracture, to improve function, and to decrease morbidity from more invasive treatments such as skin grafting.

Skin substitutes can be categorized according to whether they are acellular or cellular. Acellular products, such as cadaveric human dermis with removed cellular components, contain a scaffold or matrix of hyaluronic acid, collagen, or fibronectin. Cellular products contain living cells such as keratinocytes and fibroblasts within a matrix. These cells can be autologous, allogeneic, or from another species. Skin substitutes can be divided into three major categories: dermal replacement, epidermal replacement and dermal/epidermal replacement. They can also be used as either permanent or temporary wound coverings.

A large number of skin substitutes are commercially available or in development. Table 2 details epidermal, dermal, and combined, full-thickness skin replacements that have clinical and experimental evidence of efficacy in wound healing. Information regarding type of skin replacement, regulatory status and year of United States Food and Drug Administration (U.S. FDA) approval, product description, indications, clinical and experimental trials according to wound type, and advantages and disadvantages for each product are detailed.

Epidermal skin replacements require a skin biopsy from which keratinocytes are isolated and cultured on top of fibroblasts. Epicel^®^ (Genzyme Tissue Repair Corporation, Cambridge, MA, USA) is an epidermal skin substitute composed of cultured autogeneous keratinocytes used for permanent coverage in partial or full-thickness wounds. Laserskin**^®^** (Fidia Advanced Biopolymers, Abano Terme, Italy) is composed of autologous keratinocytes and fibroblasts cultured on a laser-microperforated biodegradable matrix of benzyl esterified hyaluronic acid.

**Table 2:** Skin substitutes for wound healing.

Epidermal Skin Replacement				
Biologic Company (FDA Approval) Product Description	Product Description	FDA Indications (Other Indications)	Clinical Trials	Advantages Disadvantages
Epicel^®^ Genzyme Tissue Repair Corporation Cambridge, MA, USA (2007) Permanent skin substitute Living Cell Therapy Cultured Epithelial Autograft (CEA)	autologous keratinocytes with murine fibroblasts are cultured to form epidermal autografts which are then processed into sheets and placed onto petroleum gauze [71]. It is used as an adjuvant to STSG or alone if STSG are not available due to the extent or severity of the burns.	*Humanitarian Device Exemption* (HDE) for treatment of deep dermal or full thickness burns (greater than or equal to 30% TBSA); grafting after congenital nevus removal (diabetic and venous ulcers)	**Burns** No RCT have been conducted to evaluate the effectiveness of this product in improving health outcomes for deep dermal/full thickness burns. In a large, single center trial, Epicel^®^ CEA was applied to 30 burn patients with a mean TBSA of 37% ± 17% TBSA. Epicel^®^ achieved permanent coverage of a mean of 26% TBSA compared to conventional autografts (mean 25%). Final CEA take was a mean 69% ± 23%. Ninety percent of these severely burned patients survived [72].	**Advantages** Use of autologous cells obviates rejection Permanent large area wound coverage, especially in extensive burns [73] **Disadvantages** Long culture time (3 weeks) Variable take rate Poor long-term results 1 day shelf life [74] Expensive Risk of blistering, contractures, and infection
Laserskin^®^ Fidia Advanced Biopolymers Abano Terme, Italy Permanent skin substitute	autologous keratinocytes and fibroblasts derived from a skin biopsy cultured on a laser-microperforated biodegradable matrix of benzyl esterified hyaluronic acid [75,76]. Cells proliferate and migrate through the matrix. Microperforations allow for drainage of wound exudate.	(diabetic foot ulcers and venous leg ulcers, partial thickness burns, vitiligo) [77,78]	**Diabetic Foot Ulcers (DFUs)** A multicenter RCT with unhealed (≥1 month) DFUs randomized 180 patients to receive intervention (Hyalograft-3D^®^ autograft and then Laserskin^®^ autograft after two weeks) or control (paraffin gauze). At 12 weeks, a 50% reduction in the intervention group was achieved significantly faster compared to control (40 *versus* 50 days). Complete ulcer healing was similar in both groups. The rate of ulcer reduction was greater in the treatment group. There was a significantly (3.65-fold) better chance of wound healing in a subgroup of hard-to-heal ulcers following autograft treatment of dorsal ulcers [79]. In a study of chronic (>6 months) foot ulcers over 15 cm^2^ in type 2 diabetic patients older than 65 years treated with Hyalograft-3D^®^ and Laserskin^®^ autograft, all ulcers healed at 12 months except for one, with a median healing time of 21 weeks [80]. In a study of 14 patients with chronic (>6 months), non-healing foot ulcers secondary to type 2 diabetes treated with Laserskin^®^ autograft, 11/14 lesions were completely healed between 7 and 64 days post-transplantation [81]. **Wounds** In a retrospective observational study in 30 patients with chronic wounds not responding to conventional therapy, keratinocytes on Laserskin^®^ to treat superficial wounds or fibroblasts on Hyalograft-3D^®^ to treat deep leg ulcers were applied; the wounds were then dressed with nanocrystalline silver dressing. A reduction in wound dimension and exudates and an increase in wound bed score was observed. The group treated with keratinocytes had a significantly greater degree of healing compared to those treated with allogenic fibroblasts [82]. Collagen matrices such as Integra^®^ have been poor recipients of cultured keratinocytes, although some studies report successes in the use of Laserskin^®^ on the neodermis of Integra^®^ after the silicone membrane is removed 14–21 days post-grafting [83,84].	**Advantages** Use of autologous cells obviates rejection Can be produced in shorterperiod of time than confluent epidermal sheets Does not require the use of the enzyme dispase to remove the sheets from culture flasks, in contrast to CEA Good graft take Low rate of infection Ease of handling during application Transparency allows woundto be visualized during dressing changes **Disadvantages** Only available in Europe 2 day shelf life Expensive
**Dermal Skin Replacement**				
**Biologic Company (FDA Approval) Product Description**	**Product Description**	**FDA Indications (Other Indications)**	**Clinical Trials**	**Advantages Disadvantages**
TransCyte^®^ Shire Regenerative Medicine, Inc. San Diego, CA, USA; Smith & Nephew, Inc., Largo, FL, USA (1997) Temporary skin substitute Composite matrix	human allogeneic fibroblasts from neonatal foreskin seeded onto silicone covered bioabsorbable nylon mesh scaffold and cultured *ex vivo* for 4–6 weeks, secreting components of the extracellular matrix and many local growth factors [85]	temporary covering of deep partial thickness and full thickness burn wounds (chronic leg ulcers (diabetic foot ulcers lasting more than 6 weeks; venous and pressure ulcers)	**Burns** 33 children with partial-thickness burn wounds were randomized to receive TransCyte^®^, Biobrane^®^, or Silvazine cream. Mean time to re-epithelization was 7.5 days, 9.5 days, and 11.2 days, respectively. Wounds requiring autografting were 5%, 17%, and 24%, respectively. TransCyte^®^ promoted faster re-epithelization, required fewer dressings, and required less autograft compared to those treated with Biobrane^®^ or Silvazine [86]. In a randomized prospective study of 21 adults with partial-thickness burn wounds to the face, patients treated with TransCyte^®^ had significantly decreased daily wound care time (0.35 ± 0.1 *versus* 1.9 ± 0.5 h), re-epithelialization time (7 ± 2 *versus* 13 ± 4 days), and pain (2 ± 1 *vs.* 4 ± 2) compared to patients treated with topical bacitracin [87]. 20 pediatric patients with TBSA over 7% were treated with TransCyte^®^ and compared to previous patients those who received standard therapy of antimicrobial ointment and hydrodebridement. Only one child required autografting in the TransCyte^®^ group, compared to 7 children in the standard treatment group. In addition, children treated with TransCyte^®^ had a significantly decreased length of stay (5.9 days) compared to those who received standard therapy (13.8 days) [88]. 110 patients with deep partial-thickness burns were treated with dermabrasion and TransCyte^®^ and compared with data from the American Burn Association Patient Registry. Patients with 0–19.9% TBSA burn treated with dermabrasion and TransCyte^®^ had a significantly shorter length of stay of 6.1 days *versus* 9.0 days. The authors compared burns of all sizes with dermabrasion and TransCyte^®^ and concluded that this method of managing partial-thickness burns reduced length of stay compared to standard care [89]. **Wounds** A randomized prospective comparison study of TransCyte^®^ and silver sulfadiazine on 11 patients with paired wound sites was performed. Wounds treated with TransCyte^®^ had significantly quicker healing times to re-epithelialization (mean 11.14 days *vs.* 18.14 days). Wound evaluations at 3, 6, and 12 months revealed that wounds treated with TransCyte^®^ healed with significantly less hypertrophic scarring than those treated with silver sulfadiazine [90].	**Advantages** Easy to remove compared to allograft Widely used for partial-thickness burns Improved healing rate 1.5 year shelf life **Disadvantages** Expensive
Dermagraft^®^ Shire Regenerative Medicine, Inc.San Diego, CA, USA (2001) Permanent or temporary skin substitute Living Cell Therapy Allogenic matrix derived from human neonatal fibroblast	cryopreserved allogenic neonatal fibroblasts derived from neonatal foreskin and cultured on bioabsorbable collagen on polyglactin (Dexon) or polyglactin-910 (Vicryl) mesh for several weeks [91]. The biodegradable mesh disappears after 3–4 weeks	Premarket approval (PMA) for full-thickness diabetic lower extremity ulcers present for longer than 6 weeks extending through the dermis but not to the tendon, muscle, or bone [92] (Chronic wounds, and noninfected wounds. It can be used as a temporary or permanent covering to support take of meshed STSG on excised burn wounds [93,94])	**DFUs** A multicenter RCT with 314 patients with chronic DFUs to Dermagraft^®^ or conventional therapy was performed. At 12 weeks, 30% of the Dermagraft^®^ patients had complete wound closure compared to 18.3% of control patients. Although the incidence of adverse events was similar for both groups, the Dermagraft group (19%) experienced significantly fewer ulcer-related adverse events (infection, osteomyelitis, cellulitis) compared to the control group (32.5%) [95]. A prospective, multicenter RCT in 28 patients with chronic DFUs (>6 weeks duration) comparing intervention (Dermagraft^®^ + saline gauze) to control (saline gauze) was performed. By week 12, significantly more DFUs healed in the intervention (71.4%) compared to the control (14.3%). Wounds closed significantly faster in patients treated with Dermagraft^®^ and the percentage of patients with wound infection was less in the Dermagraft^®^ group [96]. The DOLCE trial (ID: NCT01450943) is a randomized, single-blind, comparative trial to compare the differences among acellular matrices (Oasis^®^ (Healthpoint, Ltd Fort Worth, TX, USA), cellular matrices (Dermagraft^®^ (Shire Regenerative Medicine, Inc.), and standard of care in the treatment of DFUs using the primary outcome of complete wound closure by 12 weeks [97]. A multicenter clinical trial of Dermagraft^®^ in the treatment of DFUs in 62 patients after sharp debridement was performed. Patients received dressing changes with saline gauze or polyurethane foam dressings weekly. By week 12, 27/62 (44%) patients had complete wound closure, and 32/62 (52%) healed by week 20. Median time to healing was 13 weeks. Dermagraft^®^ was safe and effective in the treatment of non-healing DFUs [98]. A prospective multicenter randomized single-blinded study to evaluate wound healing in 50 patients with DFUs was performed. Patients were randomized into one of four groups (three separate dosages of Dermagraft^®^ and one control group). A dose response curve was observed and ulcers treated with the highest dosage of Dermagraft^®^ healed significantly more than those treated with conventional wound closure methods. 50% (6/12) of the Dermagraft^®^ and 8% (1/13) of the control ulcers healed completely. The percentage of ulcers to complete closure was significantly greater in the Dermagraft^®^ group (50% or 6/12) compared to the control group (8% or 1/13) [99]. **Venous leg ulcers** A prospective multicenter RCT to evaluate Dermagraft^®^+ compressive therapy *versus* compressive therapy alone in the treatment of venous leg ulcers was conducted. For ulcers ≤12 months duration, 49/94 (52%) patients in the Dermagraft^®^ group *versus* 36/97 (37%) patients in the control group healed at 12 weeks and this was statistically significant. For ulcers≤10 cm^2^, complete healing at 12 weeks was observed in 55/117 (47%) patients in the Dermagraft^®^ group compared with 47/120 (39%) patients in the control group, and this was statistically significant. Both groups experienced similar rates of adverse events [100]. A prospective RCT in 18 patients with venous leg ulcers treated with Dermagraft^®^ + compression therapy or compression therapy alone was performed. Healing was assessed through ulcer tracing and planimetry. The rate of healing was significantly improved in patients treated with Dermagraft^®^ [101].	**Advantages** Semitransparency allows continuous observation of underlying wound surface Cell bank fibroblasts have been tested for safety and there have been no safety issues thus far Easier to remove and higher patient satisfaction compared to allograft [94] Equivalent or better than allograft for graft take [93], wound healing time, wound exudate and infection No adverse reactions, such as evidence of rejection [93] **Disadvantages** Used for temporary coverage 6 month shelf life **Contraindications** Clinically infected ulcers Ulcers with sinus tracts Hypersensitivity to bovine products
AlloDerm^®^/Strattice^®^ LifeCell Corporation Branchburg, NJ, USA (1992) Permanent skin substituteLiving Cell Therapy Human skin allograft derived from donated human cadaver	lyophilized human acellular cadaver dermal matrix serves as a scaffold for tissue remodeling [85]	Burns, full thickness wounds [102] (breast surgery [103,104,105], soft tissue reconstruction [106])	**Burns** Three patients with full-thickness burns of the extremities were treated with AlloDerm^®^ dermal grafts followed by thin autografts. Functional performance and aesthetics were considered good to excellent [107]. The average graft take rate in 12 patients with full-thickness burn injuries in joint areas was 91.5% at one year post AlloDerm^®^ with ultrathin autograft. All patients had near normal range of motion at one year and aesthetic results were judged fair to good by both surgeons and patients [108]. **Wounds** 36 patients with oral mucosal defects reconstructed with AlloDerm^®^ grafts were evaluated. 34/36 cases (94.4%) were successfully replaced with mucosa and 2 grafts failed. Graft contraction occurred in 7/34 (20.6%) of patients with lip or buccal defects [109].	**Advantages** Immediate permanent wound coverage Allows grafting of ultra-thin STSG as one-stage procedure Template for dermal regeneration Immunologically inert since the cells responsible for immune response and graft rejection are removed during the processing Reduced scarring Can vascularize over exposed bone and tendon 2 year shelf life Good aesthetic and functional outcomes (less hypertrophic scar rates, good movement) Injectable micronized form is also available (Cymetra^®^) **Disadvantages** Risk of transmission of infectious diseases, although no cases of viral transmission have been reported No viral or prion screening Collection fluid risk (seroma, hematoma, infection) Possibility of donor rejection Expensive Requires two procedures Inability to replace both dermal and epidermal components simultaneously
Biobrane^®^ Smith & Nephew, St. Petersburg, FL, USA Temporary skin substitute Acellular matrix	acellular dermal matrix made of porcine type I collagen that is incorporated onto a porous nylon mesh with a silicone membrane. The semipermeable membrane allows for penetration of antibiotics, drainage of exudates, and control of evaporative water losses. The nylon and silicone membrane allow for adherence to the wound surface [110].	Partial thickness burns within 6 hours and donor sites of split thickness skin grafts [111] with low bacterial counts and without eschar or debris [112]; treatment of toxic epidermal necrolysis [113] and paraneoplastic pemphigus (dermabrasion, skin-graft harvesting, and laser resurfacing, chronic wounds, venous ulcers [110])	**Burns** In a retrospective chart review of children aged 4 weeks to 18 years with an average of 6% TBSA partial thickness burns, patients with Biobrane^®^ healed significantly faster compared than those treated with beta glucan collagen (9 days *vs.* 13 days). Patients requiring inpatient treatment had shorter length of hospital stay (2.6 *vs.* 4.1 days) [114] In a prospective randomized study in pediatric patients with partial thickness burns, Biobrane^®^ was compared to topical application of 1% silver sulfadiazine. Pain, pain medication requirement, wound healing time, and length of stay (LOS) were significantly reduced in the Biobrane^®^ group [115]. In a retrospective review, Biobrane^®^ promoted adherence of split thickness skin grafts to the wound, allowing fluid drainage and preventing shearing. Biobrane^®^ also facilitated healing of adjacent donor site or partial thickness burns [116]. In a controlled clinical trial of patients with partial thickness burns, compared to 1% silver sulfadiazine applied twice daily with dry gauze and elastic wraps, Biobrane^®^ decreased healing time by 29% (10.6 days *vs.* 15.0 days) and reduced pain and the use for pain medication (0.6 *vs.* 3.0 tablets) at 24 h. There was no difference in the rate of infection [117]. In a prospective study of patients with scalp defects >5 cm requiring removal of periosteum, the biosynthetic dressing was definitive in six patients and complete closure was achieved in 3.5 months [118]. In a prospective RCT of children with intermediate thickness burns with TBSA <10%, no significant difference in time to healing or pain scores were detected between use of Biobrane^®^ or Duoderm^®^, although Biobrane^®^ was more expensive [119]. In a prospective RCT of 89 children treated within 48 hours of a superficial-thickness scald burn of 5%–25% TBSA randomized to Biobrane^®^ or conservative treatment with topical antimicrobials and dressing changes, patients treated with Biobrane^®^ had significantly shorter time to healing and length of stay. There was no difference in the use of systemic antibiotics or readmission for infections [124]. In a prospective RCT comparing Biobrane^®^, Duoderm^®^, and Xeroform for 30 skin graft donor sites in 30 patients, donor sites dressed with Xeroform had a significantly shorter time to healing of 10.5 days compared to Duoderm^®^ (15.3 days) or Biobrane^®^ (19.0 days). Duoderm^®^ was reported to be the most comfortable dressing compared to Biobrane^®^ and Xeroform. Two infections developed using Biobrane^®^, one using Duoderm^®^, and none using Xeroform. Biobrane^®^ ($102.57 per patient) was the most expensive dressed compared to Duoderm^®^ ($54.88 per patient) and Xeroform ($1.16 per patient) [125].	**Advantages** Dressing naturally separates from wound Reserved for fresh wounds (<48 h) with low bacterial counts Porous material allows for exudate drainage and permeability to antibiotics Higher infection rates than other dressings [120] Reduces pain levels and nursing requirements when compared to traditional dressings [121] Shortens LOS Biobrane-L^®^ available for less aggressive adherence [122] **Disadvantages** Does not debride dead tissue [117] Permanent scarring in partial-thickness scald wounds [123]
Integra^®^ Dermal Regeneration Template (DRT) Integra Lifesciences Corporation Plainsboro, Plainsboro, NJ, USA (1996) Permanent skin substitute Acellular matrix	bilayered extracellular matrix of cross-linked bovine type 1 collagen and chondroitin-6-sulfate glycosaminoglycan dermal replacement [85,126], with a thin silicone backing which acts as a temporary epidermal substitute. The product facilitates migration of macrophages and fibroblasts to initiate angiogenesis from dermal wound bed to create granulation tissue to support graft or local tissue. Once the neo-dermis is formed, the silicone layer is removed and the wound is permanently closed with a STSG on the neo-dermis [91].	pressure ulcers, venous ulcers, diabetic ulcers, chronic vascular ulcers, surgical wounds (donor sites/grafts, post-Moh’s surgery, post-laser surgery, podiatric, wound dehiscence), trauma wounds (abrasions, lacerations, second-degree burns, and skin tears) and draining wounds (approved through 510(k) process in 2002)	**Burns** In a multicenter prospective RCT, 106 patients with life-threatening burns underwent excision and grafting. Mean burn size was 46.5% ± 15% mean TBSA. Epidermal donor sites healed 4 days sooner with Integra^®^ compared to autograft, allograft, and xenograft. There was less hypertrophic scarring with Integra^®^ [127]. Integra^®^ was applied to surgically clean, freshly excised burn wounds in 216 burn patients at 13 burn facilities in the United States. The mean total body surface area burned was 36.5%. Once the neo-dermis was generated, a thin epidermal autograft was placed. The incidence of superficial infection at Integra^®^ sites was 13.2% and of invasive infection was 3.1%. The mean take rate of Integra^®^ was 76.2% with a median of 95%. The mean take rate of epidermal autograft was 87.5% with a median take rate of 98%. This study supported the evidence that Integra^®^ is a safe and effective treatment in burn care [128]. In a prospective RCT comparing burn wounds treated with Integra^®^, STSG, and the cellulose sponge Cellonex^®^ in 10 adult patients, all products demonstrated equal histological and immunohistological findings and equal clinical appearance after one year [129]. In a RCT of 20 children with burn size ranging from 58% to 88%, there were no significant differences between Integra^®^ and control (autograft-allograft application) in burn size, mortality, and length of stay. The Integra^®^ group had lower resting energy expenditure and increased levels of serum constitutive proteins. The Integra^®^ group also had increased bone mineral content and density at 24 months and improved scarring (vascularity, pigmentation, thickness) at 12 and 24 months [130]. This study supported the use of Integra^®^ for immediate wound coverage in children with severe burns.	**Advantages** Immediate permanent skin substitute One of the most widely accepted synthetic skin substitutes Median take of 85% Two stage procedure requiring a minimum of 3 weeks between the application of Integra^®^ and STSG [131] More aesthetic compared to autograft Safe, effective, and widely utilized for burn reconstruction [128,132] Integra Flowable Wound Matrix^®^ approved through 510(k) process in 2007 **Disadvantages** Complete wound excision High risk of infection and graft loss since it is avascular [133]
		Post-excisional treatment of life threatening full thickness or deep partial thickness burn injuries [134] where autograft is not available at the time of excision or not desirable due to the condition of the patient (approved 2001); reconstruction of scar contractures when other therapies have failed or when donor sites for repair are not sufficient or desirable due to the condition of the patient; chronic lower extremity ulcers [91,92] (soft tissue defects)	**DFUs** Prospective study of patients with diabetic, non-infected plantar foot ulcers treated with Integra^®^ demonstrated complete wound closure in 7/10 patients by week 12 with no recurrent ulcers at follow-up [135]. A retrospective case studies review of five patients with DFUs with extensive soft tissue deficits and exposed bone and tendon treated with Integra^®^ followed by STSG demonstrated complete wound healing despite the failure of two grafts. No infections occured and all patients resumed ambulation [136]. **Wounds** In a retrospective study of 127 contracture releases with the application of Integra^®^ followed by epidermal autograft, 76% of the release sites, range of motion and function were rated as significantly improved or maximally improved by physicians at a mean post-operative follow-up period of 11.4 months. Patients expressed satisfaction with the results at 82% of sites. No recurrence of contracture was observed at 75% of the sites. Integra^®^ offered functional and aesthetic benefits similar to full-thickness grafts without the associated donor site morbidity [137]. Twelve patients with large wounds were randomly divided into treatment with fibrin-glue anchored Integra^®^ and postoperative negative-pressure therapy or conventional treatment. The take rate was significantly higher in the experimental treatment group (98%± 2%) compared to the conventional group (78%± 8%). The mean time from Integra^®^ application to allograft was significantly shorter in the experimental group (10 ± 1 days) compared to the conventional treatment group (24 ± 3 days), which also resulted in shorter length of stay and potentially decreased risks of complications such as infection or thrombosis [138]. With the use of dressings and STSG, Integra^®^ has been used to achieve functional and aesthetic coverage in the management of traumatic wounds of the hand with osseous, joint, or tendon exposure [139]. In a study of 31 patients who underwent Integra^®^ grafting for reconstructive surgery, complications such as silicone detachment, failure of the graft, and hematoma were observed in nine [131].	
**Epidermal/Dermal Skin Replacements (Full-Thickness)**				
**Biologic Company (FDA Approval) Product Description**	**Product Description**	**FDA Indications (Other Indications)**	**Clinical Trials**	**Advantages Disadvantages**
Apligraf^®^/Graftskin^®^ Organogenesis, Canton, MA, USA (1998, 2001) Permanent skin substitute Living Cell Therapy Composite matrix	cornified epidermal allogeneic keratinocytes derived from neonatal foreskin cultured on a type I bovine collagen gel seeded with living neonatal allogeneic human fibroblasts in dermal matrix [91]	Chronic partial and full thickness venous stasis ulcers and full thickness diabetic foot ulcers [140] (epidermolysis bullosa [141], recurrent hernia repair, pressure sores, burn reconstruction) [92]	**Venous Leg Ulcers** A Cochrane Review concluded that a bilayer artificial skin used in conjunction with compression bandaging increases venous ulcer healing compared with a simple dressing plus compression [142]. In a prospective multicenter RCT of 240 patients with hard-to-heal chronic wounds (>1 year) receiving either intervention with Graftskin^®^ plus compression or compression alone, treatment with Graftskin^®^ with compression was significantly more effective than compression therapy alone in achieving complete wound closure at 8 weeks (32% *vs.* 10%) and significantly more effective at 24 weeks (47% *vs.* 19%) [143].A previously conducted prospective RCT by the same group revealed similar results [144]. **Burns** In a multicenter RCT of 38 patients with STSG wounds, Apligraf^®^ was placed over meshed autograft while control sites were treated with meshed autograft covered with no biologic dressing or meshed allograft. There was no difference in the percent take of meshed split thickness autograft with or without Apligraf^®^. The Apligraf^®^ group demonstrated significantly improved vascularity, pigmentation, wound height and Vancouver burn scar scores, demonstrating a cosmetic and functional advantage of Apligraf^®^ compared to controls [145]. **Donor site healing** A RCT of 60 skin donor sites treated with meshed autograft, meshed Apligraf^®^, or polyurethane film dressing was conducted. The healing time with Apligraf^®^ (7.6 days) was significantly shorter than with polyurethane film dressing. In a multicenter RCT of 10 patients treated with Apligraf^®^, Apligraf^®^ dermis-only, and polyurethane film for acute STSG donor sites, there were no differences among the treatment modalities in establishing basement membrane at 4 weeks and there were no differences in other secondary outcomes [146]. **DFUs** In a multicenter RCT of 72 patients comparing Apligraf^®^ and standard therapy *versus* standard therapy alone in the treatment of DFUs, there was a significantly shorter time to complete wound closure in the Apligraf^®^ group 51.5% (17/33) compared to with standard treatment with international guidelines 26.3% (10/38) at 12 weeks [148]. In a prospective multicenter RCT of 208 patients randomly assigned to ulcer treatment with Graftskin^®^ or saline-moistened gauze (control), 63/112 (56%) of Graftskin^®^ patients achieved complete wound healing compared to 36/96 (38%) in the control at 12 weeks and this result was statistically significant. Kaplan-Meier curve to complete closure was also significantly lower for Graftskin^®^ (65 days) compared to control (90 days). Osteomyelitis and lower-limb amputations were less frequent in the Graftskin^®^ group [149]. Treatment with Apligraft^®^ plus good wound care for DFUs results in 12% reduction in costs during first year of treatment compared to good wound care alone [150]. **Wounds** In a prospective RCT of 31 patients requiring full-thickness surgical excision for non-melanoma skin cancer, patients were randomized to receive a single application of Apligraf^®^ or to heal by secondary intention. Apligraf^®^ reduced post-operative pain in this setting, but it was not determined whether it could decrease healing time or result in better aesthetic outcomes [151]. In a prospective controlled clinical trial, 48 deep dermal wounds were created and Apligraf^®^, STSG, or dressing was applied. Apligraf^®^ demonstrated more cellular infiltrate but less vascularization compared to controls. Apligraf^®^ demonstrated survival of allogeneic cells in acute wounds for up to six weeks and was recommended for the management of acute surgical wounds [152].	**Advantages** Small wounds require one application Improved cosmetic (scar tissue, pigmentation, texture) and functional outcomes in chronic wounds [145] Primary role in treating chronic ulcers **Disadvantages** Large wounds may require multiple applications 5 day shelf life [91] Expensive Potential for viral transmission; mothers blood and donor’s cells screened; cell banks screened for product safety Consider ethics with use of biological material: bovine collagen (Hindus, Buddhists; vegetarians); derived from foreskin (Quakers) [147] **Contraindications** Infected wounds Allergy to bovine collagen
OrCel^®^ Forticell Bioscience, New York City, NY, USA (1998) Living Cell Therapy Composite matrix	neonatal foreskin derived keratinocytes and dermal fibroblasts cultured in separate layers into a type I bovine collagen porous sponge [85]. During healing, autologous skin cells replace the cells in the product.	Approved for HDE in 2001 for use in patients with dystrophic epidermolysis bullosa undergoing hand reconstruction surgery to close and heal wounds created by surgery, including donor sites; PMA approval for autograft donor sites in burn patients (overlay on split thickness skin grafts to improve cosmesis and function) [92] (chronic diabetic and venous wounds)	A randomized matched pairs study comparing treatment of split-thickness donor site wounds with OrCel^®^ or Biobrane-L^®^ revealed that scarring and healing times for sites treated with OrCel^®^ were significantly shorter than for sites treated with Biobrane-L^®^ [153].	**Advantages** 9 month shelf life **Disadvantages** Cryopreserved Cannot be used in infected wounds, in patients who are allergic to any animal products, or in patients allergic to penicillin, gentamycin, streptomycin, or amphotericin B
GraftJacket^®^ Wright Medical Technology, Inc., Arlington, TX, USA, licensed by KCI USA, Inc., San Antonio, TX, USA Permanent skin substitute Human skin allograft derived from donated human cadaver	micronized acellular human dermis with a dermal matrix and intact basement membrane to facilitate ingrowth of blood vessels	(deep and superficial wounds, sinus tract wounds, tendon repair, such as rotator cuff repair) [154] not subject to FDA pre-notification approval as it is a human cell or tissue based product	**DFUs** Multicenter, retrospective study in the treatment of 100 chronic, full thickness wounds of the lower extremity in 75 diabetic patients revealed a 91% healing rate and suggested its use in the treatment of complex lower extremity wounds. No significant differences were observed for matrix incorporation or complete healing. Mean time to complete healing was 13.8 weeks [155]. In a prospective multicenter RCT comparing GraftJacket^®^ with standard of care therapies for the treatment of DFUs in 86 patients for 12 weeks, the proportion of completely healed ulcers between the groups was statistically significant. The odds of healing in the study group were 2.7 times higher than in the control group. The odds of healing were 2.0 times higher in the study group than in the control group when adjusted for ulcer size at presentation [156]. A prospective randomized study evaluating diabetic patients with lower extremity wounds demonstrated that patients treated with GraftJacket^®^ healed significantly faster than those with conventional treatment at 1 month [157]. A prospective single center RCT comparing intervention (sharp debridement + GraftJacket^®^+ mineral oil-soaked compression dressing) to control (wound gel with gauze dressing) for the treatment of full-thickness chronic non-healing lower extremity wounds in 28 diabetic patients revealed that at 16 weeks, 12/14 patients treated with GraftJacket^®^ had complete wound closure compared to 4/14 patients in the control group. Significant differences were observed for wound depth, volume, and area [158]. In a prospective, randomized single blind pilot study of 40 patients with debrided diabetic lower extremity wounds, GraftJacket^®^ was compared to the hydrogel wound dressing Curasol^®^. At 4 weeks, there was a significant reduction in the ulcer size in the GraftJacket^®^ group compared to debridement only. At 12 weeks, 85% of the patients with GraftJacket^®^ healed compared to 5% of controls [157]. A retrospective multicenter series in 12 patients with DFUs and complex, deep, irregularly-shaped, tunneling sinus tracts treated with GraftJacket Xpress Scaffold^®^ (a micronized, decellularized flowable soft tissue scaffold that can be delivered through a syringe into the wound cavity) demonstrated complete healing in 10/12 patients at 12 weeks [159]. In a prospective case series of 17 patients with debrided, non-infected, non-ischemic, neuropathic DFUs treated with a single application of GraftJacket^®^ with weekly silicone dressing changes, 82.5% of wounds had complete re-epithelialization in 20 weeks, with a mean time to healing of 8.9 ± 2.7 weeks [160].	**Advantages** 2 year shelf life Pre-meshed for clinical application Single application Utilized in both deep and superficial wound healing **Disadvantages** Cryopreserved
PermaDerm^®^ Regenicin, Inc., Little Falls, NJ, USA Permanent skin substitute	autologous keratinocytes and fibroblasts cultured on bovine collagen scaffold	Orphan status approval as a permanent skin substitute in burns	No clinical trials available.	**Advantages** No risk of rejection Permanent substitute for massive burn injury **Disadvantages** No clinical trials or long-term studies available

Dermal skin replacements provide greater stability to the wound and prevent the wound from contracting. Transcyte^®^ (Shire Regenerative Medicine, Inc., San Diego, California, USA; Smith & Nephew, Inc., Largo, FL, USA) is composed of human allogeneic fibroblasts from neonatal foreskin seeded onto silicone covered bioabsorbable nylon mesh scaffold and cultured *ex vivo* for 4–6 weeks [85]. Transcyte^®^ is often used as a non-living, temporary wound covering for partial- and full-thickness burns after excision [161]. A derivative of Transcyte^®^ is Dermagraft^®^ (Shire Regenerative Medicine, Inc., San Diego, California, USA), a skin substitute composed of living allogenic fibroblasts incorporated into a bioresorbable polyglactin mesh that secretes extracellular matrix (ECM) proteins, collagen, growth factors and cytokines into the wound site in the provision of viable living dermal substitute [162,163]. Dermagraft^®^ has shown improvement in the treatment of chronic diabetic foot ulcers. AlloDerm^®^/Strattice^®^ (LifeCell Corporation, Branchburg, NJ, USA) are lyophilized human acellular cadaver dermal matrices which serve as a scaffold for tissue remodeling. Autologous keratinocytes may be seeded and cultured on Alloderm^®^ to form an epithelium; together; these can be utilized for wound and burn closure. Subsequent to its administration to a wound site, AlloDerm^®^ is shown to exhibit cellular infiltration and neovascularization [164]. Biobrane^®^ (Smith & Nephew, St. Petersburg, FL, USA) is a synthetic dermis temporary skin substitute composed of inner nylon and outer silicone with bovine collagen used for temporary coverage in partial and full-thickness burns. Integra^®^ Dermal Regeneration Template (DRT) (Integra Lifesciences Corporation, Plainsboro, NJ, USA) is an example of a composite skin graft. It is composed of an outer layer of silicone and a cross-linked bovine type I collagen glycosaminoglycan dermal matrix. Once the dermal layer has regenerated, the silicone layer is removed and the wound is permanently closed with a split thickness skin graft (STSG) on the neo-dermis. Integra^®^ is used for permanent coverage of full-thickness burns when combined with thin skin graft.

Epidermal/Dermal skin replacements are also called as full-thickness skin substitutes and are composed of both epidermal and dermal layers. Autologous or allogeneic fibroblasts and keratinocytes are used in their preparation. The allogenically derived Apligraf^®^ (Organogenesis, Canton, MA, USA) is a bilayered matrix construct similar to a microscopic skin layer. Specifically, it is comprised of a lower dermal layer of bovine type 1 collagen combined with human fibroblasts (extracted from postnatal foreskin) and an upper layer that consists of human keratinocytes, along with granulocyte/macrophage colony-stimulating factors. Apligraf^®^ has been used for permanent coverage of non-healing chronic wounds (such as diabetic foot ulcers), surgical wounds, pressure wounds, neuropathic wounds and venous insufficiency ulcers. Apligraf^®^ has been observed *in vitro* to generate extracellular matrix structural elements and modulators inclusive of tissue inhibitors of matrix metalloproteinases and glycoprotein fibronectin [2]. OrCel^®^ (Forticell Bioscience, New York, NY, USA) is a composite matrix composed of keratinocytes and dermal fibroblasts cultured in separate layers into a type I bovine collagen porous sponge. It is used in patients with dystrophic epidermolysis bullosa undergoing hand reconstruction surgery and at autograft donor sites in burn patients [92]. GraftJacket^®^ (Wright Medical Technology, Inc., Arlington, TX, USA, licensed by KCI USA, Inc., San Antonio, Texas, USA), is an acellular derivative of human dermis. GraftJacket^®^ was shown to facilitate accelerated healing and initiate depth and volume reductions in wounds [156]. PermaDerm^®^ (Regenicin, Inc., Little Falls, NJ, USA) is a newer product that acts as a permanent skin substitute to cover large burns. It is composed of autologous keratinocytes and fibroblasts cultured on bovine collagen scaffold [165].

### 3.2. Contraindications

Biological skin equivalents such as allogenically derived Apligraf^®^ or Dermagraft^®^ have an existing, albeit significantly low, risk of disease transmission due to their allogenicity [162]. In the case of Apligraf^®^, it has been verified in a number of studies that the cells it delivers are not sustained within the wound site beyond six weeks, and has inconsistent effects on the wound basement membrane, *in vivo* collagen composition and vascularization [2,146,152].

### 3.3. Clinical Trial Based Evidence

Greer *et al.* [166] compared a number of advanced wound therapies in the treatment of ulcers in regard to the proportion of ulcers healed and time to healing. This study reviewed randomized controlled trials from the literature (MEDLINE 1995–2013, Cochrane Library, and existing systemic reviews), which involved patients who were typically middle-aged white males. The 56 trials encompassed lower extremity or foot ulcers, with 35 cases of patients with diabetic ulcers, 20 patients with venous ulcers, and one patient with arterial ulcers. The duration of therapies generally spanned from 4 to 20 weeks, with a mean ulcer duration from 2 to 94 weeks. The mean ulcer size ranged from 1.9 to 41.5 cm^2^. Of the advanced wound care products used in these trials, the biological skin equivalent Apligraf^®^ demonstrated moderate-strength evidence for enhanced healing, as did negative pressure wound therapy. Low-strength evidence was shown for platelet-derived growth factors and silver cream in comparison to standard care. For arterial ulcers, there was an improvement in healing with biological skin equivalent. Although the evidence was deemed as limited, the conclusion of the authors was that several advanced wound care therapies appeared to enhance the number of ulcers healed, as well as to reduce the times for healing. 

A clinical randomized, double-blind, standard-controlled study was undertaken, which compared burn wounds that were treated with silver zinc sulfadiazine cream (control) against those treated with the identical cream that also contained silk sericin. The study involved 29 patients presenting with 65 burn wounds that covered at least 15% of total body surface areas. It was observed that the typical time for attaining 70% re-epithelialization in the sericin group was approximately 5–7 days shorter than the control group. The control group required 29.28 ± 9.27 days for complete burn wound healing, while the sericin group attained this condition within 22.42 ± 6.33 days with no indication of severe reaction or infection in any wound [49].

Multiple clinical trials have been conducted with the living skin equivalents Apligraf^®^ and Dermagraft^®^. A retrospective controlled trial was undertaken that involved 2517 patients (446 Apligraf^®^, 1892 Regranex^®^ (a human platelet-derived growth factor topical gel for the treatment of lower extremity diabetic neuropathic ulcers), 125 platelet releasates, 54 combined) and found that diabetic foot ulcers initially treated with Apligraf^®^ were 31.2% more likely to heal than those administered with topical growth factor and 40% more likely to heal than those treated with platelet releasates [95]. In a prospective, randomized controlled trial involving 72 patients (33 Apligraf^®^, 39 with saline moistened gauze control), it was found that at 12 weeks, full wound closure was observed in 51.5% (17 of 33) of Apligraf^®^ patients in contrast to 26.3% (10 of 38) of control patients [148]. An additional prospective, randomized controlled trial involved 74 patients (38 autograft + Apligraf^®^, 36 autograft alone or + allograft) with dull and partial thickness burns. It was found at 22 months that 58% of the Apligraf^®^ sites were deemed of better quality than the controls, with 26% as equivalent and 16% as worse. Further, Apligraf^®^ treated patients (47%) exhibited normal vascularity in contrast to 6% of control patients [145].

A prospective, randomized controlled trial with Dermagraft^®^ studied 314 patients (130 Dermagraft^®^, 115 controls) with diabetic foot ulcers. At 12 weeks, 30% of the Dermagraft^®^ patients were healed in comparison to 8.3% of the control patients, who were treated with standard wet-to-dry dressings [95]. An additional prospective, randomized controlled trial was undertaken with 18 patients (10 Dermagraft^®^, eight controls) with venous ulcers, which revealed that the healing rate after 12 weeks was enhanced considerably in those patients treated with Dermagraft^®^ + compression (five patients (50%)) as opposed to compression on its own (one patient (12.5%)). In addition, the perfusion of capillaries in the Dermagraft^®^ group increased by 20%, in comparison to 4.9% in the compression group [101].

## 4. Biomembranes for Wound Healing

### 4.1. Description

Biocompatible vegetal biomembranes of natural rubber/latex, amniotic, polyurethane and poly-dl-lactic acid (PDLLA) comprise a class of versatile interventions for the treatment and healing of wounds. Additionally, biomembranes may be impregnated with a wide range of bioactive compounds to further facilitate and promote wound healing.

### 4.2. Mechanism and Indications

Human amniotic membranes, such as Biomembrane**^®^** (Matrix Company, Ismailia, Egypt) are comprised of skin-like fetal ectoderm, consisting of four layers (epithelial, basement membrane, connective tissue fibroblasts, and spongy layer), which have demonstrated angiogenic properties. The membrane is freeze dried to 5% water content and then gamma irradiated (25 kgy) to ensure sterilization. These biomembranes exhibit a 1000-fold improvement in efficacy over split-thickness human skin grafts, though the specific mechanisms remain unclear [167,168]. Further, amniotic membranes are found to inhibit the alpha smooth muscle protein actin, resulting in a significant reduction in the generation of scar tissue in comparison to a moist wound dressing control [169]. Additional benefits included decreased pain, protection from infection and control of the loss of electrolytes and albumin.

The polyurethane film, Tegaderm^TM^ (3M, Saint Paul, MN, USA), exhibits gas semi-permeability, which acts to augment the rate of epithelialization. This may be due the retention of carbon dioxide, which translates to sustaining a low pH. The pain relief that is reportedly associated with this film may be the result of the exclusion of atmospheric oxygen, which negates the generation of prostaglandin E2, via the oxygen-reliant cyclo-oxygenase system [167,170]. An additional imparted benefit secondary to the semi-permeability of Tegaderm^TM^ is the regulation of transforming growth factor beta (TGF-β) via the mediation of transepidermal water transfer [171]. It also stimulates the propagation of keratinocytes through the activation of integrins a5 and a6 to encourage enhanced and rapid wound healing [172].

A biocompatible vegetal biomembrane derived from the *Hevea brasiliensis* rubber tree exhibited the capacity to initiate angiogenesis and re-epithelialization in the chronic ulcers of diabetic patients. Its activity in the healing process appears most prominent at the inflammatory stage, where the microenvironment is transformed by robust angiogenesis followed by re-epithelialization [173].

A non-toxic, biocompatible, biodegradable, and non-carcinogenic crosslinked gelatin hydrogel biomembrane was developed for use as a wound dressing via the addition of a naturally occurring genipin crosslinking agent, and compared to a glutaraldehyde-crosslinked control. The resulting genipin infused biomembrane exhibited considerably less inflammation along with more rapid re-epithelialization and subsequent wound healing than the control, which may have been facilitated by a lower level of genipin imparted cytotoxicity [36].

### 4.3. Contraindications

Despite stringent preparation protocols, there might be a very low risk of bacterial or viral transmission via the use of human amniotic membranes on open wounds.

### 4.4. Clinical Trial Based Evidence

Adly *et al.* [167] conducted a randomized, controlled clinical trial to compare the efficacy of an amniotic membrane (Biomembrane^®^) *group I* (23 patients) and a polyurethane membrane (Tegaderm^TM^) dressing *group II* (23 patients) in the treatment of burns (scald and flame). There were no notable differences between the two groups. The criteria were inclusion of both genders and all age groups with <50% total body surface area affected with either second or third degree burns. The *group I* patients exhibited a considerably lower infection rate (one patient (4.3%) in *group I* compared to three patients (13.0%) in *group II*) and required fewer dressing changes than *group II* (highest dressing change frequency was once per day in 30.4% of *group I* patients, in comparison to five times per day in 60.9% of *group II* patients). In addition, electrolyte disturbance was evident in 17.4% of patients in *group I*, compared with 60.9% of patients in *group II*. Albumin loss was indicated in 39.1% of patients in *group I* in contrast to 60.9% of patients in *group II.* In terms of pain and healing times, 43.5% of *group I* patients experienced pain during dressing, compared with 60.9% in *group II*. Healing frequency was 47.8% (11–20 days) for *group I* in contrast to 39.1% in *group II* spanning the same time period.

## 5. Scaffolds for Wound Healing

### 5.1. Description

Hybrid scaffolds comprised of polymeric substrates coated with bioactive materials, collagen, silk fibroin, as well as advanced tissue engineered substrates impregnated with endothelial progenitor cells, and nanomaterial-based scaffolds may be employed as advanced wound dressings to initiate and expedite wound healing.

### 5.2. Mechanisms and Indications

Collagen is a component of the extracellular matrix, which has found established utility as a biomaterial in cell therapies and tissue engineering via the provision of a viable substrate for the attachment and propagation of cells. In the treatment of wounds, collagen scaffolds offer a feasible platform for the topical conveyance of cells into the wound bed, increase the healing of wounds and initiate angiogenesis and neovasculogenesis.

O’Loughlin *et al.* [174] investigated the use of type 1 collagen scaffolds for the topical delivery of autologous circulating angiogenic (CACs) cells (precursors to endothelial progenitor cells), to full thickness cutaneous ulcers. It was revealed that the CACs could also be pre-stimulated through the addition of matricellular protein osteopontin (OPN), a glycoprotein involved in immune function, neovascularization, and facilitation of cell migration and survival [175]. The inclusion of OPN served to augment wound healing. It was demonstrated that scaffolds comprised of type 1 collagen, which has been shown to sustain angiogenesis [176], when infused with CACs and enhanced with OPN, resulted in the formation of larger diameter blood vessels than untreated wounds, and thus acceleration of the wound healing process [174]. 

Ehashi *et al.* [177] compared subcutaneously implanted scaffolds for their host body reactions in order to assess their wound healing capacities. The scaffolds consisted of collagen coated porous (Ø32 μm and Ø157 μm) polyethylene (CCPE), bio-inert poly(2-methacryloyloxyethyl phosphorylcholine-co-n-butyl methacrylate) (PMB) coated polyethylene, and uncoated porous polyethylene (UPPE) (control). Subsequent to their immersion in sterile solution for an appropriate period, six samples (two of each type with different pore diameters) were implanted under the skins of mouse models, and then resected after seven days. In terms of vascularization, it was observed that small vessels were induced on the UPPE, albeit contingent on the pore size (more activity seen with Ø32 μm pores than Ø157 μm pores). Interestingly, the reverse was true for the CCPE, with more activity seen with the Ø157 μm pore sample. There was no vessel growth activity associated with the PMB scaffolds. A deoxyribonucleic acid (DNA) microarray assay was then employed to conduct genetic analyses, which showed that the CCPE scaffold had a more highly distributed level of gene expression than did the PMB scaffold. The PMB scaffold showed the up-regulation of genes associated with the suppression of inflammation. The CCPE scaffold indicated up-regulation of genes related to inflammation, angiogenesis, and wound healing. The authors concluded that the up-regulation of interleukin-1b and angiogenesis associated genes within the porous scaffolds likely contributed to the mediation of tissue regeneration.

A novel scaffold comprised of electrospun core-shell gelatin/poly(l-lactic acid)-co-poly-(ε-caprolactone) nanofibers, which encapsulated a photosensitive polymer poly (3-hexylthiophene) (P3HT) and epidermal growth factor (EGF) at its core, was investigated by Jin *et al.* [178] as a potential skin graft. It was found that fibroblast propagation was activated under exposure to light in contrast to its absence and cells akin to keratinocytes were found only on the light exposed scaffolds. The researchers propose that these light sensitive nanofibers may have utility as a unique scaffold for the healing of wounds and the reconstitution of skin.

Bacterial (or microbial) cellulose has been investigated by Fu *et al.* [179] for its capacity to enable wound healing and skin tissue rejuvenation. Specific bacteria are involved in the biosynthesis of this natural polymer, which has unique properties in contrast to plant based cellulose, encompassing biocompatibility, hydrophilicity, high water retention, elasticity, transparency, conformability and the capacity for absorbing wound generated exudate during inflammation. These features position microbial cellulose to have great potential for biomedical advancements in skin tissue repair.

### 5.3. Contraindications

Scaffolds that are comprised of hyaluronan (an anionic polysaccharide), even though non-cytotoxic and biodegradable, may disrupt cell adhesion and the regeneration of tissues due to its hydrophilic surface properties [177]. Additional drawbacks for tissue engineered scaffolds in the case of severe burns relate to their unreliable adhesion to lesions and failure to replace dermal tissues [180].

### 5.4. Clinical Trial Based Evidence

The clinical performance of bacterial cellulose (BC) scaffold Dermafill^TM^ (AMD/Ritmed, Tonawanda, New York, USA) wound dressings (*Acetobacter xylinum* derived) was assessed by Portal *et al.* [181] who compared the reduction in wound size of chronic non-healing lower extremity ulcers following standard care. A total of 11 chronic wounds were evaluated for the time required to achieve 75% epithelization, by comparing non-healing ulcers prior to and following the application of Dermafill^TM^. The median observation timeline for chronic non-healing wounds under standard care prior to the application was 315 days. When BC scaffolds were applied to these same wounds, the median time to 75% epithelization was decreased to 70 days. Thus, the authors concluded that BC scaffold-initiated wound closure for non-healing ulcers proceeded considerably more rapidly than did standard care wound dressings.

Morimoto *et al.* [182] investigated the clinical efficacy of a unique synthetic collagen/gelatin sponge (CGS) scaffold for the treatment of chronic skin ulcers. This artificial dermal scaffold demonstrated the capacity to sustainably release basic fibroblast growth factor (bFGF) over 10 days or longer. One of the criteria for the study group was the inclusion of chronic skin ulcers that had not healed over a time period of at least four weeks. These wounds treated with CGS, which was infused with 7 or 14 μg/cm^2^ of bFGF following debridement, and assessed two weeks subsequent to their application. Positive improvement in the wound beds was defined by the emergence of granulated and epithelialized areas of 50% or greater. Out of a total of 17 subjects, it was observed that 16 showed wound bed improvements, with no discernable difference between the low and high dose groups. There was rapid recovery from mild adverse reactions.

## 6. Conclusions

The healing of surface and deep wounds of the epidermis is a complex multistage process, but one that may nevertheless be expedited utilizing strategies such as the application of active biologic, biomembrane or scaffold based wound dressings. Specific therapeutic compounds and cell species including epidermal stem cells may be utilized to impregnate biocompatible and/or biodegradable substrates, including membranes and scaffolds to facilitate rapid revascularization, re-epithelialization, and healing of wound beds.

## Abbreviations

RNA: Ribonucleic Acid
IL-6: Interleukin 6
TNF-α: Tumor Necrosis Factor Alpha
LTC4: Leukotriene C4
TXB2: Thromboxane B2
UVB: Ultraviolet B
MIF: Migration Inhibitory Factor
NO: Nitric Oxide
RCT: Randomized Controlled Trial
TBSA: Total Body Surface Area
STSG: Split-Thickness Skin Graft
COX-2: Cyclooxygenase-2
IL-1β: Interleukin-1 beta
NF-κB: Nuclear Factor kappa-light-chain-enhancer of activated B cells
IL-10: Interleukin 10
ATP: Adenosine Triphosphate
KATP: Potassium Channels
CEA: Cultured Epithelial Autograft
HDE: Humanitarian Device Exemption
DFU: Diabetic Foot Ulcers
PMA: Premarket Approval
LOS: Length of Stay
TGF-β: Transforming Growth Factor-beta
CAC_s_: Circulating Angiogenic Cells
OPN: Osteopontin
CCPE: Collagen Coated Porous Polyethylene
PMB: Poly(2-methacryloyloxyethyl phosphorylcholine-co-n-butyl methacrylate)
UPPE: Uncoated Porous Polyethylene
DNA: Deoxyribonucleic Acid
P3HT: Photosensitive Polymer Poly (3-hexylthiophene)
CGS: Collagen/Gelatin Sponge
